# Early machine learning prediction of hospitalized patients at low risk of respiratory deterioration or mortality in community-acquired pneumonia: Derivation and validation of a multivariable model

**DOI:** 10.17305/bb.2023.9754

**Published:** 2024-04-01

**Authors:** Yewande E Odeyemi, Amos Lal, Erin F Barreto, Allison M LeMahieu, Hemang Yadav, Ognjen Gajic, Phillip Schulte

**Affiliations:** 1 Division of Pulmonary and Critical Care Medicine, Mayo Clinic, Rochester, MN, United States; 2 Department of Pharmacy, Mayo Clinic, Rochester, MN, United States; 3 Division of Clinical Trials and Biostatistics, Mayo Clinic, Rochester, MN, United States

**Keywords:** Community-acquired pneumonia (CAP), machine learning, predictive modeling, advanced respiratory support, mortality

## Abstract

Current prognostic tools for pneumonia predominantly focus on mortality, often neglecting other crucial outcomes such as the need for advanced respiratory support. The objective of this study was to develop and validate a tool that predicts the early risk of non-occurrence of respiratory deterioration or mortality. We conducted a single-center, retrospective cohort study involving hospitalized adult patients with community-acquired pneumonia (CAP) and acute hypoxic respiratory failure from January 2009 to December 2019 (*n* ═ 4379). We employed the gradient boosting machine (GBM) learning to create a model that estimates the likelihood of patients requiring advanced respiratory support (high-flow nasal cannula [HFNC], non-invasive mechanical ventilation [NIMV], and invasive mechanical ventilation [IMV]) or mortality during hospitalization. This model utilized readily available data, including demographic, physiologic, and laboratory data, sourced from electronic health records and obtained within the first 6 h of admission. Out of the cohort, 890 patients (25.2%) either required advanced respiratory support or died during their hospital stay. Our predictive model displayed superior discrimination and higher sensitivity (cross-validation *C*-statistic ═ 0.71; specificity ═ 0.56; sensitivity ═ 0.72) compared to the pneumonia severity index (PSI) (*C*-statistic ═ 0.65; specificity ═ 0.91; sensitivity ═ 0.24; *P* value < 0.001), while maintaining a negative predictive value (NPV) of approximately 0.85. These data demonstrate that our machine-learning model predicted the non-occurrence of respiratory deterioration or mortality among hospitalized CAP patients more accurately than the PSI. The enhanced sensitivity of this model holds the potential for reliably excluding low-risk patients from pneumonia clinical trials.

## Introduction

Pneumonia remains a common cause of acute hypoxemic respiratory failure that requires hospitalization, with significant morbidity and mortality when the intensive care unit (ICU) transfer is delayed [1]. Current prognostic and risk stratification tools for community-acquired pneumonia (CAP) primarily focus on mortality prediction, aiming to inform on illness severity and the initial site of care. However, there is limited evidence regarding the disease-specific prediction of deterioration during a patient’s hospital stay [2]. The pneumonia severity index (PSI) identifies patients with low risk of mortality more accurately than other simple prognostic tools, such as the confusion, urea, respiratory rate, blood pressure, and 65 years of age or older (CURB-65) score, the confusion, respiratory rate, blood pressure, and 65 years of age or older (CRB-65) score, and the age, dehydration, respiratory failure, orientation disturbance, and low blood pressure (A-DROP) score. Therefore, it is effective and safe in guiding the initial site of care (whether outpatient or inpatient) with broad generalizability and reproducibility [3–8]. Prediction of mortality however does not provide accurate identification of patients who would benefit from intensified management strategies once they are hospitalized [9]. This subset of high-risk patients has been defined as having severe CAP when ICU admission is the sole clinical surrogate [10]. Other prognostic scoring tools, including the 2001 American Thoracic Society (ATS), 2007 Infectious Disease Society of America (IDSA)/ATS, and the systolic blood pressure, multilobar chest radiography, albumin level, respiratory rate, tachycardia, confusion, oxygen level, and pH level (SMART-COP) score, have performed better than the PSI in predicting ICU admission. However, these tools either directly or indirectly consider criteria that inherently reflect critical disease, disregarding the trajectory of the need for advanced respiratory support such as high-flow nasal cannula (HFNC) or non-invasive mechanical ventilation (NIMV), whether inside or outside the ICU setting [10–13]. The objective of this study was to develop a tool using machine learning methods for the early risk prediction of non-occurrence of respiratory deterioration or mortality in hospitalized CAP patients. Such a tool could be useful for prognostic enrichment in clinical trials of CAP interventions by excluding low-risk patients [14].

## Materials and methods

### Source of data and participants

A retrospective cohort from a single center was analyzed, comprising hospitalized adult patients (aged ≥ 18 years) with CAP from January 2009 to December 2019. The cohort was used to develop the model for respiratory deterioration, using routinely and readily available information from electronic health records. This encompassed demographic data, clinical features, and laboratory data obtained within the initial 6-h post-admission. Patients who denied the utilization of their medical records for research purposes were excluded (10%). The design and reporting of this observational study adhered to the guidelines specified by the Transparent Reporting of a multivariable prediction model for Individual Prognosis Or Diagnosis (TRIPOD).

CAP was defined as an acute infection of the lung parenchyma that is associated with clinical symptoms (cough, fever, pleuritic chest pain, and dyspnea) and a new radiographic infiltrate, not acquired in the hospital or healthcare setting, identified by the International Classification of Diseases (ICD) 9 (481–486) and 10 (J13, J15, and J18) codes and note search. The exclusion criteria were similar to other studies [15] and they included: lack of research authorization, prior hospitalization within the 15 days leading up to the admission, aspiration pneumonia, hospital/ventilator-acquired pneumonia (if diagnosed after 48-h post-admission), interstitial lung disease, leukopenia, neutropenia, acquired immunodeficiency syndrome (AIDS), and human immunodeficiency virus (HIV) infection.

### Outcomes

The primary outcome encompassed a combination of the requirement for advanced respiratory support (which includes the use of HFNC, NIMV, and invasive mechanical ventilation [IMV]) or mortality during hospitalization. Given that this study focused on in-hospital decompensation, we excluded patients who were not hospitalized and those without a need for supplemental oxygen.

The secondary outcomes of interest were hospital mortality, the need for IMV, and the need for NIMV.

For these specified outcomes, patients who have already met that status within the first 6 h of admission were excluded from further analysis.

### Predictors

Predictor variables included age, sex, race, height, weight, blood pressure, heart rate, respiratory rate, temperature, medical comorbidities (such as congestive heart failure, chronic obstructive pulmonary disease [COPD], asthma, liver disease, neoplastic disease, and renal disease), laboratory data (white blood cell count with differentials [neutrophils, eosinophils, lymphocytes], bicarbonate, sodium, blood urea nitrogen [BUN], and blood gases), and clinical scores (PSI, Sequential Organ Failure Assessment [SOFA] score, and Acute Physiology and Chronic Health Evaluation [APACHE] III score), obtained within initial 6 h of admission. For predictors measured repeatedly or longitudinally within these 6 h, only the first observation was used. For analysis purposes, race was grouped as White or other/unknown.

### Sample size

**Figure 1.**
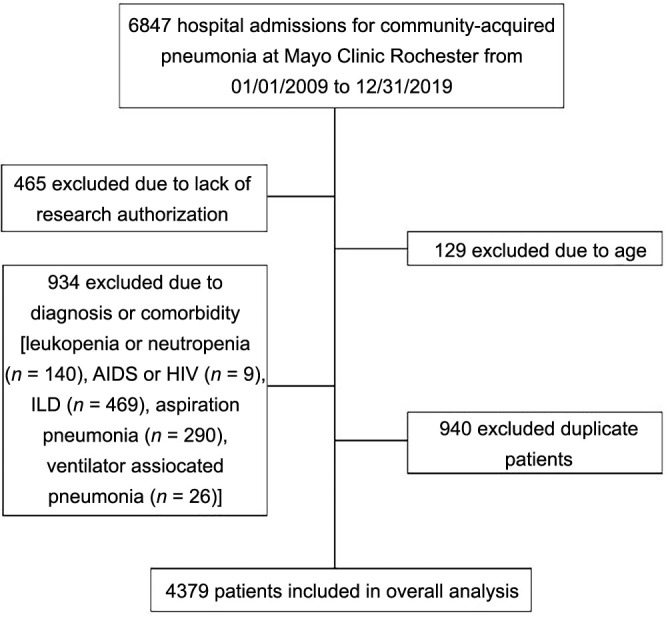
**Study flow diagram.** AIDS: Acquired immunodeficiency syndrome; HIV: Human immunodeficiency virus; ILD: Interstitial lung disease.

All eligible patients (*n* ═ 4379) meeting the criteria were included (Figure 1). For our primary outcome, which was the need for advanced respiratory support or mortality, 3528 patients had not reached that status at 6-h post-admission. To develop a predictive model for the need of advanced respiratory support or mortality, we established that a sample size of *n* ═ 3528 would be expected to produce a model with a mean absolute prediction error of 0.028 in the predicted outcome probabilities. This was based on an outcome rate of 25.2% and the inclusion of 32 predictor variables in the model [16]. Consequently, our sample was expected to produce a model with predicted values that would exhibit a small mean error when applied to new individuals.

### Ethical statement

The Mayo Clinic Institutional Review Board (IRB) approved this study prior to its initiation (IRB number: 17-011140, modification approval date: June 2021, Title: Concordant versus discordant corticosteroid use with markers of inflammation in critically ill patients with pneumonia and ARDS). Informed consent was waived, and all procedures conformed to the ethical standards set by the Mayo Clinic IRB and the Helsinki Declaration of 1975.

### Statistical analysis

Patient demographics, physiological parameters, and clinical and laboratory data are presented using the median (IQR) for continuous variables and frequency (percentage) for categorical variables.

To test the hypothesis that a combination of patient characteristics will accurately predict the need for advanced respiratory support or mortality, stochastic gradient boosting machine (GBM) learning was employed. A 5-fold repeated cross-validation (ten repeats) was used with a grid-search approach to select tuning parameters [17]: shrinkage, interaction depth, minimum number of observations in the terminal nodes, bag fraction, and number of trees. A threshold level for classification to advanced respiratory support or mortality was selected to maximize sensitivity to a negative predictive value (NPV) of at least 0.85. This approach was chosen over using Youden’s Index or other ad hoc methods because the anticipated successful model would aim to screen out those at low risk (of needing advanced respiratory support or death) as a prognostic enrichment strategy for enrollment in clinical trials. Primary metrics for model development and validation included the area under the receiver operating characteristic curve (*C*-statistic). Metrics for threshold classification included sensitivity, specificity, positive predictive value (PPV), and NPV.

GBM models were also evaluated for secondary outcomes, which were in-hospital mortality, and the need for IMV and NIMV, using the same methods applied for the primary outcome. Those prediction models were developed using the subset of patients who were event-free at the prediction time (within 6 h of admission).

Our model was additionally tuned to exclude variables with less influence for the primary and secondary outcome (Model 1: all variables, Model 2: parsimonious model). DeLong’s test was used to compare the area under the curve (AUC) scores for our parsimonious models against PSI and CURB-65 for the outcomes of advanced respiratory support or mortality, and solely for mortality.

Any missing predictors were treated as a distinct and possibly informative segment of the data, reflecting actual practices where such omissions are common. This allows the resulting GBM prediction model to handle missing (unmeasured) inputs and still produce the predicted probability of an event. There were no missing data for the primary and secondary outcomes or for the following predictors: age, gender, race, comorbidities, altered mental status, PSI, or CURB-65. Data management and analysis were conducted using SAS version 9.4 (SAS Institute Inc., Cary, NC, USA) and R version 4.1.2 (RStudio Team 2021, Boston, MA, USA). We used the R packages “gbm” and “caret” for model training.

## Results

Demographics and clinical characteristics are described in Table S1. Patient outcomes within the cohort are detailed in Table 1. Of the cohort, a total of 890 patients (25.2%) needed advanced respiratory support or died in the hospital.

**Table 1:** Patient outcomes of interest

	**Overall (*n* ═ 4379)**
Advanced respiratory support or mortality during hospitalization, *n* (%)*	890/3528 (25%)
Hospital mortality, *n* (%)	155 (3%)
Invasive mechanical ventilation, *n* (%)*	402/3966 (10%)
Non-invasive mechanical ventilation, *n* (%)*	674/3819 (18%)

*Based on the sample size of patients who were event-free at 6-h post-admission.

### Primary outcome

**Figure 2.**
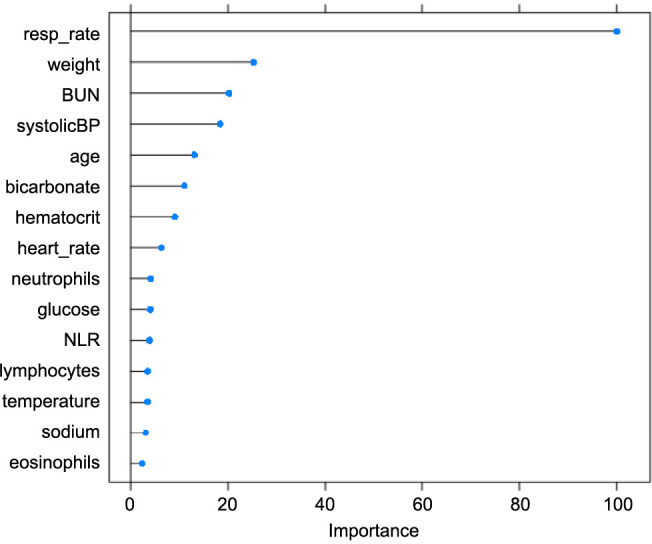
**Relative importance of the top 15 variables in the advanced respiratory support or mortality model.** Resp: Respiratory; BUN: Blood urea nitrogen; BP: Blood pressure; NLR: Neutrophil to lymphocyte ratio.

**Figure S1.**
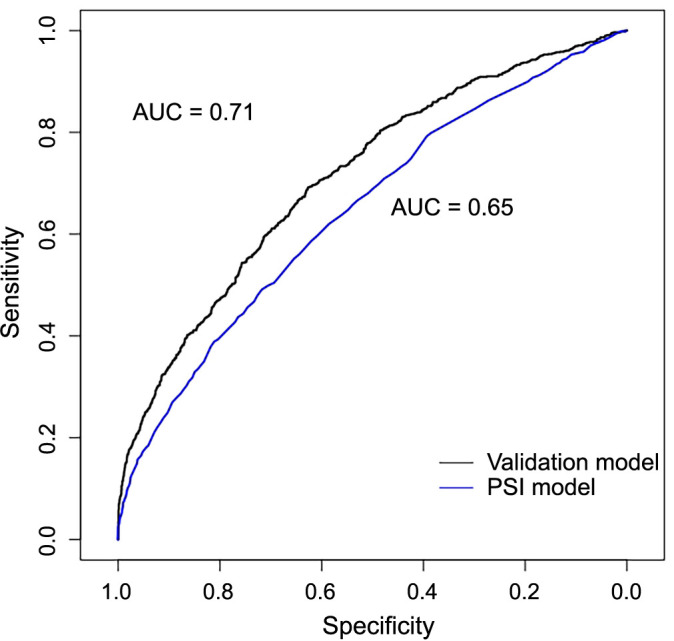
**ROC plot comparing the validation model with the PSI model for advanced respiratory support or mortality.** ROC: Receiver operating characteristic; PSI: Pneumonia severity index; AUC: Area under the curve.

The GBM prediction model of the need for advanced respiratory support or mortality due to pneumonia demonstrated a fair discrimination (cross-validation *C*-statistic ═ 0.713; accuracy rate ═ 61.2%, 95% CI 59.5%–62.8%; NPV ═ 0.860; specificity ═ 0.574; sensitivity ═ 0.723). For classification purposes, a predicted probability of 0.30 or above was deemed as “at risk” to achieve an NPV ≥ 0.85. In contrast, the PSI’s performance in predicting advanced respiratory support was poor (cross-validation *C*-statistic ═ 0.647; accuracy rate ═ 74.2%, 95% CI 72.7%–75.6%; NPV ═ 0.780; specificity ═ 0.911; sensitivity ═ 0.238) (Table 2 and Figure S1). Similarly, the CURB-65’s performance was also poor (cross-validation *C*-statistic ═ 0.621; accuracy rate ═ 38.1%, 95% CI 36.5%–39.7%; NPV ═ 0.850; specificity ═ 0.209; sensitivity ═ 0.891). Upon further tuning to omit less influential variables (for a parsimonious model, Table S2) and incorporating exploratory models that include the need for vasopressor support, the results remained consistent. According to DeLong’s test, our GBM model had a better AUC score compared to PSI (*z* score ═ 5.19; *P* value < 0.001) and CURB-65 (*z* score ═ 7.15; *P* value < 0.001). Variables with the highest importance in the final model were respiratory rate, weight, BUN, and systolic blood pressure (Figure 2).

**Table 2:** Prediction of advanced respiratory support or mortality

	**Gradient boosting models with Bernoulli distribution**					
	**AUC score**	**Accuracy rate (95% CI)**	**Specificity**	**Sensitivity**	**NPV**	**PPV**
*First model*						
Cross-validation model	0.710	0.600 (0.584 – 0.617)	0.560	0.722	0.857	0.356
*Second model (parsimonious)*						
Cross-validation model	0.713	0.612 (0.595 – 0.628)	0.574	0.723	0.860	0.364
PSI cross-validation model	0.647	0.742 (0.727 – 0.756)	0.911	0.238	0.780	0.475
CURB-65 cross-validation model	0.621	0.381 (0.365 – 0.397)	0.209	0.891	0.850	0.275

AUC: Area under the curve; NPV: Negative predictive value; PPV: Positive predictive value; PSI: Pneumonia severity index; CURB-65: Confusion, urea, respiratory rate, blood pressure, and 65 years of age or older score.

### Secondary outcomes

**Figure S2.**
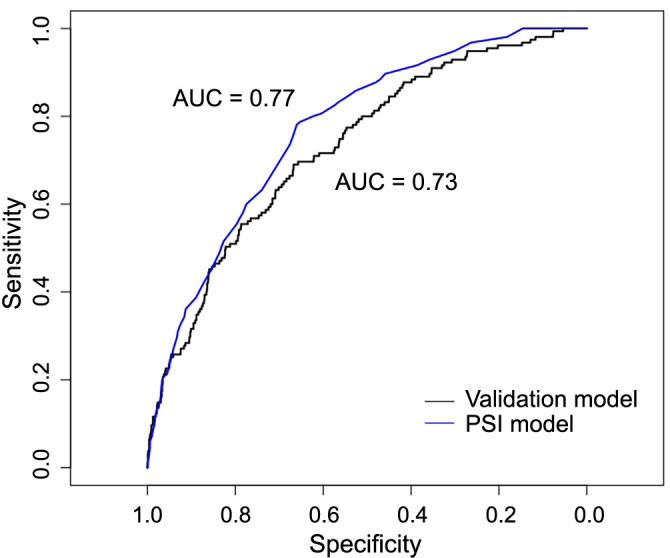
**ROC plot comparing the validation model with the PSI model solely for mortality.** ROC: Receiver operating characteristic; PSI: Pneumonia severity index; AUC: Area under the curve.

During hospitalization, 155 patients (3.5%) died. The model’s prediction of in-hospital mortality demonstrated an acceptable discrimination in the dataset, with a cross-validation AUC score of 0.752 (accuracy rate ═ 95.9%, 95% CI 95.3%–96.5%; NPV ═ 0.967; specificity ═ 0.991; sensitivity ═ 0.084). This was slightly lower than the PSI, which had a cross-validation AUC score of 0.772 (accuracy rate ═ 96.5%, 95% CI 95.9%–97.0%; NPV ═ 0.965; specificity ═ 1.000; sensitivity ═ 0) (Table 3 and Figure S2).

**Table 3:** Prediction of mortality

	**Gradient boosting models with Bernoulli distribution**					
	**AUC score**	**Accuracy rate (95% CI)**	**Specificity**	**Sensitivity**	**NPV**	**PPV**
*First model*						
Cross-validation model	0.731	0.959 (0.953 – 0.965)	0.991	0.084	0.967	0.265
*Second model (parsimonious)*						
Cross-validation model	0.727	0.963 (0.957 – 0.968)	0.995	0.077	0.967	0.364
PSI cross-validation model	0.772	0.965 (0.959 – 0.970)	1.000	0	0.965	–
CURB-65 cross-validation model	0.695	0.965 (0.959 – 0.970)	1.00	0	0.965	–

AUC: Area under the curve; NPV: Negative predictive value; PPV: Positive predictive value; PSI: Pneumonia severity index; CURB-65: Confusion, urea, respiratory rate, blood pressure, and 65 years of age or older score.

**Figure 3.**
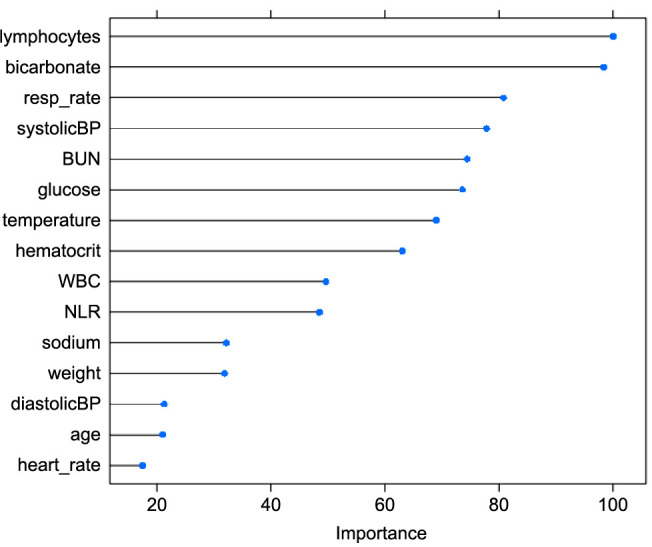
**Relative importance of the top 15 variables in the mortality model.** Resp: Respiratory; BUN: Blood urea nitrogen; BP: Blood pressure; WBC: White blood cells; NLR: Neutrophil to lymphocyte ratio.

The CURB-65 prediction of in-hospital mortality was similar to our GBM model, with a cross-validation AUC score of 0.695 (accuracy rate ═ 96.5%, 95% CI 95.9%–97.0%; NPV ═ 0.965; specificity ═ 1.000; sensitivity ═ 0). DeLong’s test, when comparing our GBM model, did not detect significant differences in the AUC score compared to the PSI (*z* score ═ −1.66; *P* value ═ 0.096) and the CURB-65 (*z* score ═ 1.39; *P* value ═ 0.164). The variables of highest importance in the final model included lymphocytes, bicarbonate, respiratory rate, and systolic blood pressure (Figure 3 and Table S3).

After the exclusion of patients who required invasive and non-invasive ventilation within 6 h of admission, 402 patients (10.1%) and 674 patients (17.7%) patients required IMV and NIMV, respectively, during hospitalization. The model’s predictions for the need for IMV (cross-validation AUC ═ 0.736; accuracy rate ═ 87.0%, 95% CI 85.9%–88.0%; NPV ═ 0.924; specificity ═ 0.932; sensitivity ═ 0.321), and for the need for NIMV (cross-validation AUC ═ 0.732; accuracy rate ═ 66.3%, 95% CI 64.8%–67.8%; NPV ═ 0.892; specificity ═ 0.673; sensitivity ═ 0.619) were deemed acceptable.

## Discussion

Although the PSI is effective and safe in guiding the initial site of care (whether inpatient or outpatient treatment), it poorly predicts which hospitalized patients might require intensified management [2, 9]. Other prognostic tools, such as the IDSA/ATS guidelines, SMART-COP, and early warning scores, have shown better performance in predicting ICU admissions based on clinical endpoints of IMV and/or vasopressor support [10, 11–13]. However, these tools focus on a critical late stage of the disease course, where the endpoint of ICU admission identifies only a specific subgroup of high-risk patients. Moreover, ICU admission decisions are prone to multiple biases, including limited resources, advance directives, and hospital policies.

Importantly, these prognostic tools do not consider the need for advanced respiratory support methods, such as HFNC and NIMV, which are increasingly being used in both the ICU and non-ICU settings.

Therefore, it is important to explore clinical endpoints other than ICU admission and mortality when aiming for early prognostication of hospitalized patients with CAP.

Predicting the need or the lack of need for advanced respiratory support (HFNC/NIMV/IMV), early in the disease’s course can provide valuable insights. Such predictions, unlike the clinical endpoints of HFNC/NIMV failure described in studies like those involving the ratio of oxygen saturation/FiO_2_ to respiratory rate (ROX) index and the heart rate, acidosis, consciousness, oxygenation, and respiratory rate (HACOR) score [19–22], could potentially inform important research enrichment strategies. Such prediction tools could facilitate prognostic enrichment in clinical trials by excluding low-risk patients who are unlikely to benefit from an intervention.

In our large single-center cohort study of patients with CAP, we found that early prediction (within the first 6 h of admission) of hospitalized patients at low risk of respiratory deterioration or mortality was better using a machine-learning model compared to the PSI and the CURB-65. The PSI’s higher specificity would classify more patients as low risk, while distinguishing poorly those at high risk due to its lower sensitivity. Nevertheless, with the same NPV, our model’s higher sensitivity, compared to the PSI, assures a reduction in misclassification of high-risk patients as low-risk, thereby facilitating the absolute exclusion of those at low risk. While these findings may have limited clinical relevance, an *R* shiny application for the model is in development for use as a prognostic enrichment tool. This will help exclude low-risk patients in time-sensitive pneumonia clinical trials.

In our study, the machine-learning models for secondary outcomes showed a fair discriminatory performance when compared to the primary outcome. The model’s prediction of in-hospital mortality was not statistically different compared to the PSI in this cohort, and the AUC was consistent with prior studies [23–25]. Interestingly, the most important variables in the model included the bicarbonate and the absolute lymphocyte count, both of which are readily available but not included in the PSI. When comparing our findings with a recent machine-learning model developed to predict 30-day mortality in CAP patients, our model showed a lesser discriminatory performance compared to the causal probabilistic network (CPN) (AUC ═ 0.80) [26]. The CPN model utilized data collected within the first 24 h of admission, in contrast to our model which utilized data within the first 6 h of admission. For similar reasons, the model’s prediction of the need for IMV was acceptable but weaker when compared to the SMART-COP (AUC ═ 0.87) [13]. The model’s prediction of the need for NIMV also showed acceptable discriminatory capacity. To our understanding, no other study has reported similar findings utilizing data obtained within the first 6 h of admission to predict the need for NIMV during hospitalization.

The use of continuous variables rather than dichotomous variables, which can sometimes oversimplify variable interpretation, and the application of a 5-fold cross-validation are notable strengths of our study. However, several limitations also need to be highlighted including the potential bias existing within the dataset, inherent to its single-center nature. As a result, the findings presented in this paper are limited to the characteristics as seen in a large academic referral center. In this study, we excluded patients with mild ambulatory diseases and those who did not require oxygen in the first 6 h of admission. This exclusion was related to our specific cohort of interest. Additionally, the routinely measured clinical variables within the first 6 h of admission, which were less likely to be missing, could be insufficient for optimal model discrimination while unmeasured parameters, including but not limited to treatment interactions, genetic predisposition, and pathogen characteristics unknown at the time of admission, may have an important role in the risk of respiratory deterioration or mortality. Lastly, our study did not account for the potential occurrence of a second, independent pneumonia event or deaths unrelated to pneumonia.

Additional research is needed to evaluate the prediction of pneumonia-specific clinical endpoints in CAP, beyond just mortality, intubation needs, and ICU admissions, in order to better identify patients who are more or less likely to deteriorate shortly after being admitted.

## Conclusion

Our findings demonstrate that a machine-learning model more accurately predicted the absence of respiratory deterioration or mortality among hospitalized CAP patients compared to the PSI. The model’s higher sensitivity could help in effectively excluding low-risk patients from pneumonia clinical trials.

## Acknowledgments

We would like to acknowledge the Anesthesia Clinical Research Unit study coordinator, Alberto Marquez for his help with data extraction.

## Supplemental data

**Table S1:** Demographics and clinical characteristics of patients

**Characteristic**	**Overall (*n* ═ 4379)**
*Sex, n (%)*	
Female	2015 (46%)
Male	2364 (54%)
Age (years), median (IQR)	73.6 (61.2 – 83.3)
*Race, n (%)*	
American Indian/Alaskan Native	19 (0%)
Asian	51 (1%)
Black or African American	67 (2%)
Other	85 (2%)
Unknown	28 (1%)
White	4129 (94%)
*Comorbidities, n (%)*	
Asthma	664 (15%)
Congestive heart failure	1334 (30%)
COPD	1565 (36%)
Liver disease	138 (3%)
Neoplastic disease	1649 (38%)
Renal disease	1294 (30%)
*First physical examination, median (IQR)*	
Altered mental status, *n* (%)	534 (12%)
Weight (kg), *n* ═ 4054	78.7 (64.7 – 95.6)
Diastolic BP (mmHg), *n* ═ 4316	69.0 (59.0 – 81.0)
Pulse (bpm), *n* ═ 4288	91.0 (78.0 – 105.0)
Respiratory rate (rpm), *n* ═ 2789	21.0 (18.0 – 25.0)
Systolic BP (mmHg), *n* ═ 4316	127.0 (112.0 – 144.0)
Temperature (^∘^C), *n* ═ 4279	36.8 (36.6 – 37.2)
*Lab and radiologic findings, median (IQR)*	
Blood urea nitrogen (mg/dL), *n* ═ 4158	20.0 (14.0 – 30.0)
Glucose (mg/dL) , *n* ═ 4192	129.0 (108.0 – 165.0)
Hematocrit (%), *n* ═ 4198	37.5 (33.3 – 41.2)
Sodium (mmol/L), *n* ═ 4212	137.0 (134.0 – 140.0)
Partial pressure of arterial oxygen (mmHg), *n* ═ 1615	68.0 (47.0 – 92.0)
WBC (x 10^9/L), *n* ═ 4201	11.8 (8.6 – 15.8)
Neutrophils (x 10^9/L), *n* ═ 3701	9.5 (6.6 – 13.2)
Eosinophils (x 10^9/L), *n* ═ 2602	0.1 (0 – 0.2)
Lymphocytes (x 10^9/L), *n* ═ 3689	1.0 (0.6 – 1.5)
Neutrophil/Lymphocyte ratio, *n* ═ 3682	9.7 (5.4 – 17.0)
Bicarbonate (mmol/L), *n* ═ 3982	25.0 (23.0 – 28.0)
Arterial pH, *n* ═ 1766	7.4 (7.3 – 7.4)
Pleural effusion, *n* ═ 635, n (%)	326 (51%)
*Initial clinical scores, median (IQR)*	
Pneumonia severity index	115.0 (89.0 – 143.0)
CURB-65	3.0 (2.0 – 3.0)
SOFA, *n* ═ 1812	4.0 (2.0 – 7.0)
APACHE III, *n* ═ 1812	50.0 (39.0 – 64.0)

COPD: Chronic obstructive pulmonary disease; BP: Blood pressure; bpm: Beats per minute; rpm: Respirations per minute; WBC: White blood cells; CURB-65: Confusion, urea, respiratory rate, blood pressure, and 65 years of age or older score; SOFA: Sequential Organ Failure Assessment score; APACHE III: Acute Physiology and Chronic Health Evaluation III score.

**Table S2:** Variables used in the predictive models for advanced respiratory support or mortality

**Variable**	**Model 1**	**Model 2**	**PSI**
*Demography*			
Age	Yes	Yes	Yes
Sex	Yes		Yes
Race	Yes	Yes	
Nursing home resident			Yes
*Comorbidities*			
Neoplastic disease	Yes		Yes
Liver disease	Yes		Yes
Congestive heart failure	Yes	Yes	Yes
Renal disease	Yes		Yes
Asthma	Yes	Yes	
COPD	Yes		
Cerebrovascular disease			Yes
*Vital parameters*			
Weight	Yes	Yes	
Temperature	Yes	Yes	Yes
Systolic blood pressure	Yes	Yes	Yes
Diastolic blood pressure	Yes	Yes	
Heart rate	Yes	Yes	Yes
Respiratory rate	Yes	Yes	Yes
Mental status	Yes	Yes	Yes
*Lab parameters*			
BUN	Yes	Yes	Yes
Sodium	Yes	Yes	Yes
Glucose	Yes	Yes	Yes
Hematocrit	Yes	Yes	Yes
WBC	Yes	Yes	
Eosinophils	Yes	Yes	
Neutrophils	Yes	Yes	
Lymphocytes	Yes	Yes	
NLR	Yes	Yes	
Bicarbonate	Yes	Yes	
pH			Yes
PaO2			Yes
*Radiology*			
Chest X-ray			Yes

PSI: Pneumonia severity index; COPD: Chronic obstructive pulmonary disease; BUN: Blood urea nitrogen; WBC: White blood cells; NLR: Neutrophil to lymphocyte ratio.

**Table S3:** Variables used in the predictive models for mortality

**Variable**	**Model 1**	**Model 2**	**PSI**
*Demography*			
Age	Yes	Yes	Yes
Sex	Yes	Yes	Yes
Race	Yes		
Nursing home resident			Yes
*Comorbidities*			
Neoplastic disease	Yes	Yes	Yes
Liver disease	Yes	Yes	Yes
Congestive heart failure	Yes	Yes	Yes
Renal disease	Yes	Yes	Yes
Asthma	Yes	Yes	
COPD	Yes	Yes	
Cerebrovascular disease			Yes
*Vital parameters*			
Weight	Yes	Yes	
Temperature	Yes	Yes	Yes
Systolic blood pressure	Yes	Yes	Yes
Diastolic blood pressure	Yes	Yes	
Heart rate	Yes	Yes	Yes
Respiratory rate	Yes	Yes	Yes
Mental status	Yes	Yes	Yes
*Lab parameters*			
BUN	Yes	Yes	Yes
Sodium	Yes	Yes	Yes
Glucose	Yes	Yes	Yes
Hematocrit	Yes	Yes	Yes
WBC	Yes	Yes	
Eosinophils	Yes	Yes	
Neutrophils	Yes	Yes	
Lymphocytes	Yes	Yes	
NLR	Yes	Yes	
Bicarbonate	Yes	Yes	
pH			Yes
PaO2			Yes
*Radiology*			
Chest X-ray	Yes		Yes
Care level at 6 h post admission	Yes		

PSI: Pneumonia severity index; COPD: Chronic obstructive pulmonary disease; BUN: Blood urea nitrogen; WBC: White blood cells; NLR: Neutrophil to lymphocyte ratio.

## References

[1] Phua J, Ngerng WJ, Lim TK (2010). The impact of a delay in intensive care unit admission for community-acquired pneumonia. Eur Respir J.

[2] Fine MJ, Auble TE, Yealy DM, Hanusa BH, Weissfeld LA, Singer DE (1997). A prediction rule to identify low-risk patients with community-acquired pneumonia. N Engl J Med.

[3] Aujesky D, Fine MJ (2008). The pneumonia severity index: a decade after the initial derivation and validation. Clin Infect Dis.

[4] Atlas SJ, Benzer TI, Borowsky LH, Chang Y, Burnham DC, Metlay JP (1998). Safely increasing the proportion of patients with community-acquired pneumonia treated as outpatients: an interventional trial. Arch Intern Med.

[5] Marrie TJ, Lau CY, Wheeler SL, Wong CJ, Vandervoort MK, Feagan BG (2000). A controlled trial of a critical pathway for treatment of community-acquired pneumonia. JAMA.

[6] Carratalà J, Fernández-Sabé N, Ortega L, Castellsagué X, Rosón B, Dorca J (2005). Outpatient care compared with hospitalization for community-acquired pneumonia: a randomized trial in low-risk patients. Ann Intern Med.

[7] Yealy DM, Auble TE, Stone RA, Lave JR, Meehan TP, Graff LG (2005). Effect of increasing the intensity of implementing pneumonia guidelines: a randomized, controlled trial. Ann Intern Med.

[8] Renaud B, Coma E, Labarere J, Hayon J, Roy P-M, Boureaux H (2007). Routine use of the pneumonia severity index for guiding the site-of-treatment decision of patients with pneumonia in the emergency department: a multicenter, prospective, observational, controlled cohort study. Clin Infect Dis.

[9] Kolditz M, Ewig S, Höffken G (2013). Management-based risk prediction in community-acquired pneumonia by scores and biomarkers. Eur Respir J.

[10] Mandell LA, Wunderink RG, Anzueto A, Bartlett JG, Campbell GD, Dean NC (2007). Infectious Diseases Society of America/American Thoracic Society consensus guidelines on the management of community-acquired pneumonia in adults. Clin Infect Dis.

[11] Ewig S, Ruiz M, Mensa J, Marcos MA, Martinez JA, Arancibia F (1998). Severe community-acquired pneumonia. Assessment of severity criteria. Am J Respir Crit Care Med.

[12] Chalmers JD, Mandal P, Singanayagam A, Akram AR, Choudhury G, Short PM (2011). Severity assessment tools to guide ICU admission in community-acquired pneumonia: systematic review and meta-analysis. Intensive Care Med.

[13] Charles PGP, Wolfe R, Whitby M, Fine MJ, Fuller AJ, Stirling R (2008). SMART-COP: a tool for predicting the need for intensive respiratory or vasopressor support in community-acquired pneumonia. Clin Infect Dis.

[14] Wong HR (2017). Intensive care medicine in 2050: precision medicine. Intensive Care Med.

[15] Barlow G, Nathwani D, Davey P (2007). The CURB65 pneumonia severity score outperforms generic sepsis and early warning scores in predicting mortality in community-acquired pneumonia. Thorax.

[16] Riley RD, Ensor J, Snell KIE, Harrell FE, Martin GP (2020). Calculating the sample size required for developing a clinical prediction model. Bmj.

[17] Moons KG, Altman DG, Reitsma JB, Ioannidis JP, Macaskill P, Steyerberg EW (2015). Transparent reporting of a multivariable prediction model for Individual Prognosis or Diagnosis (TRIPOD): explanation and elaboration. Ann Intern Med.

[18] Zhou HJ, Lan TF, Guo SB (2020). Outcome prediction value of National Early Warning Score in septic patients with community-acquired pneumonia in emergency department: a single-center retrospective cohort study. World J Emerg Med.

[19] Prakash J, Bhattacharya PK, Yadav AK, Kumar A, Tudu LC, Prasad K (2021). ROX index as a good predictor of high flow nasal cannula failure in COVID-19 patients with acute hypoxemic respiratory failure: a systematic review and meta-analysis. J Crit Care.

[20] Ricard JD, Roca O, Lemiale V, Corley A, Braunlich J, Jones P (2020). Use of nasal high flow oxygen during acute respiratory failure. Intensive Care Med.

[21] Roca O, Caralt B, Messika J, Samper M, Sztrymf B, Hernández G (2019). An index combining respiratory rate and oxygenation to predict outcome of nasal high-flow therapy. Am J Respir Crit Care Med.

[22] Duan J, Han X, Bai L, Zhou L, Huang S (2017). Assessment of heart rate, acidosis, consciousness, oxygenation, and respiratory rate to predict noninvasive ventilation failure in hypoxemic patients. Intensive Care Med.

[23] Aujesky D, Auble TE, Yealy DM, Stone RA, Obrosky DS, Meehan TP (2005). Prospective comparison of three validated prediction rules for prognosis in community-acquired pneumonia. Am J Med.

[24] Buising KL, Thursky KA, Black JF, MacGregor L, Street AC, Kennedy MP (2006). A prospective comparison of severity scores for identifying patients with severe community acquired pneumonia: reconsidering what is meant by severe pneumonia. Thorax.

[25] Capelastegui A, España PP, Quintana JM, Areitio I, Gorordo I, Egurrola M (2006). Validation of a predictive rule for the management of community-acquired pneumonia. Eur Respir J.

[26] Cilloniz C, Ward L, Mogensen ML, Pericàs JM, Méndez R, Gabarrús A, et al.

